# High level of HIV-1 drug resistance among patients with HIV-1 and HIV-1/2 dual infections in Guinea-Bissau

**DOI:** 10.1186/s12985-015-0273-9

**Published:** 2015-03-11

**Authors:** Sanne Jespersen, Martin Tolstrup, Bo Langhoff Hønge, Candida Medina, David da Silva Té, Svend Ellermann-Eriksen, Lars Østergaard, Christian Wejse, Alex Lund Laursen, for The Bissau HIV cohort study group

**Affiliations:** Bandim Health Project, Indepth Network, Apartado 861, Bissau, Guinea-Bissau; Department of Infectious Diseases, Aarhus University Hospital, Palle Juul-Jensens Boulevard 99, 8200 Aarhus N, Denmark; Department of Clinical Immunology, Aarhus University Hospital, Palle Juul-Jensens Boulevard 99, 8200 Aarhus N, Denmark; National HIV Programme, Ministry of Health, Bissau, CP 1013 Bissau, Guinea-Bissau; Department of Clinical Microbiology, Aarhus University Hospital, Palle Juul-Jensens Boulevard 99, 8200 Aarhus N, Denmark; GloHAU, Center for Global Health, School of Public Health, Aarhus University, Bartholins Allé 2, 8000 Aarhus C, Denmark

**Keywords:** HIV-1, HIV-1/2 dual infection, Sub-Saharan Africa, Drug resistance, Antiretroviral treatment, Guinea-Bissau

## Abstract

**Background:**

With the widespread use of antiretroviral treatment (ART) in Africa, the risk of drug resistance has increased. The aim of this study was to evaluate levels of HIV-1 resistance among patients with HIV-1 and HIV-1/2 dual infections, treated with ART, at a large HIV clinic in Guinea-Bissau.

**Findings:**

Patients were selected from the Bissau HIV cohort. All patients had HIV-1 or HIV-1/2 dual infection, a CD4 cell count performed before and 3–12 months after starting ART, and a corresponding available plasma sample. We measured viral load in patients with HIV-1 (n = 63) and HIV-1/2 dual (n = 16) infections a median of 184 days after starting ART (IQR: 126–235 days). In patients with virological failure (defined as viral load >1000 copies/ml) and with sufficient plasma available, we performed an HIV-1 genotypic resistance test. Thirty-six patients (46%) had virological failure. The CD4 cell count did not predict treatment failure. Of the 36 patients with virological failure, we performed a resistance test in 15 patients (42%), and nine patients (9/15; 60%) had resistance mutations. The most common mutation was K103N, which confers high-level resistance to non-nucleoside reverse transcriptase inhibitors (NNRTI). No major mutations against protease inhibitors (PI) were found.

**Conclusions:**

Our results showed that patients with HIV-1 and HIV-1/2 dual infections in Guinea-Bissau had a high rate of virological failure and rapid development of NNRTI resistance. It remains to be determined whether a more robust, PI-based treatment regimen might benefit this population more than NNRTIs.

## Findings

Widespread use of antiretroviral treatment (ART) in Africa has increased the risk of drug resistance [1]. Factors that contribute to drug resistance include lack of plasma viral load monitoring [2], treatment interruptions due to drug stocking discontinuities [3], and drug interactions [4].

Most patients in Africa initiate ART with two nucleoside/nucleotide reverse transcriptase inhibitors (NRTIs) and one non-nucleoside reverse transcriptase inhibitor (NNRTI) [5]. Africans have a high risk of developing the K103N NNRTI mutation, which is connected to poor adherence, due to a common genetic polymorphism that causes slow plasma NNRTI clearance and functional NNRTI monotherapy, when treatment is interrupted [6].

The West African country, Guinea-Bissau, has the highest HIV-2 prevalence worldwide [7-9]. HIV-2 is naturally resistant to NNRTIs [10], hence, patients with HIV-2 or HIV-1/2 dual infections must be treated with a protease inhibitor (PI)-based regimen. Differences in HIV-1 and HIV-2 resistance patterns may lead to complex drug resistance challenges for ART options in HIV-1/2 dual infections. This study is the first to report data on HIV resistance in Guinea-Bissau among patients with HIV-1 and HIV-1/2 dual infections. Based on data from neighboring countries, we suggest that HIV resistance may be a substantial problem [11-13].

## Methods

This retrospective, follow-up study accessed data from a clinical HIV cohort at Hospital Nacional Simão Mendes, in Bissau, the capital of Guinea-Bissau [14].

Whenever a CD4 cell count is performed, surplus plasma is stored in a biorepository in Aarhus, Denmark. From this repository, we identified data for adult patients with HIV-1 or HIV-1/2 dual infections that had CD4 cell counts and stored plasma samples acquired before and after 3–12 months of ART. HIV-1/HIV-2 discrimination was performed with a SD Bioline HIV 1/2 3.0 test (Standard Diagnostics Inc, Kyonggi-do, South Korea). All stored plasma from patients with HIV-1/2 dual infections underwent an immunofluorescence discriminatory HIV-test (INNO-LIA; Innogenetics, Ghent, Belgium) [15]. When INNO-LIA and Bioline produced divergent results, INNO-LIA was considered the gold standard.

HIV-1 viral load was measured at the Department of Clinical Microbiology, Aarhus University Hospital, Denmark, with COBAS® AmpliPrep/COBAS® TaqMan® (Roche Diagnostics GmbH, Mannheim, Germany). The lower limit of detection was 20 copies/ml. Virological failure was defined as a viral load >1000 copies/ml [5].

When sufficient plasma was available, we studied HIV-1 genotypic resistance in patients with virological failure by sequencing the protease and reverse transcriptase genes with ViroSeq® 2.0 (Abbott Laboratories, Illinois, USA). Mutations were classified as minor or major according to ART resistance consensus statements from the Stanford HIV RT and Protease Sequence database [16]. Subtype classifications were extracted from the Stanford database.

We used the Chi-square test for categorical variables to compare characteristics of patients with HIV-1 and HIV-1/2 dual infections, and patients with or without virological failure. We compared continuous variables with the Wilcoxon rank-sum test (non-normal distributions). The significance level was set at 0.05. Statistical analyses were performed with Stata IC 11.0 (StataCorp, College Station, Texas, USA).

All patients provided voluntary, signed, dated, informed consent, or fingerprints when illiterate, prior to enrolment into the cohort. The ongoing HIV cohort studies were approved by the national ethics committee of Guinea-Bissau (Parecer NCP/No.15/2007).

## Results

Viral load was measured in stored plasma samples from patients with HIV-1 (n = 63) and HIV-1/2 dual (n = 16) infections, acquired before and 3 to 12 months after (median 184 days, interquartile range (IQR): 126–235 days) starting ART (Table 1). No patient was pregnant and no information was available regarding ART during previous pregnancies.

**Table 1:** **Patient characteristics**

**Characteristic**	**Number of patients (%), unless other indicated (total N = 79)**
Sex	
Females	47 (59)
Males	32 (41)
Age at inclusion, median years (IQR)	36 (28–43)
HIV-type	
HIV-1	63 (80)
HIV-1/2	16 (20)
Baseline CD4 cell count, median cells/μl (IQR)	134 (62–207)
Baseline viral load, median copies/ml (IQR)	73,473 (3,798-264,033)
Baseline ART	
2 NRTIs + 1 NNRTI	53 (67)
2 NRTIs + 1 PI	23 (29)
3 NRTIs	3 (4)
Post ART CD4 cell count, median cells/μl (IQR)	217 (157–310)
Post ART viral load, median copies/ml (IQR)	203 (0–25.153)

*IQR: Interquartile range.*

**Figure 1:**
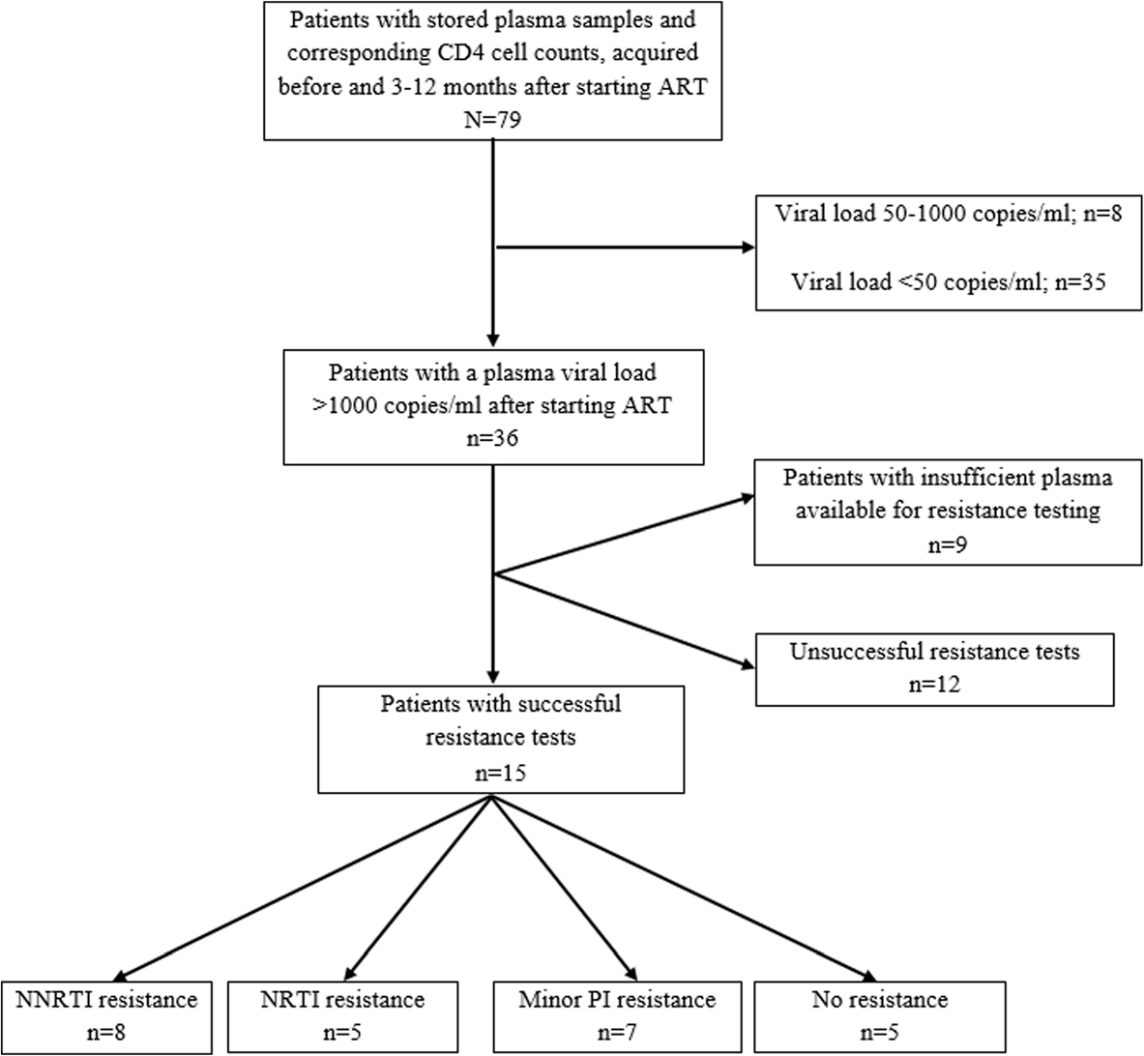
**Flow chart of patient selection.** ART: antiretroviral treatment; NNRTI: non-nucleoside reverse transcriptase inhibitors; NRTI: nucleoside/nucleotide reverse transcriptase inhibitors; PI: protease inhibitors.

Patients with HIV-1 and HIV-1/2 dual infections showed no significant differences in HIV-1 viral load (85,373 vs. 39,555 copies/ml, p = 0.37) or CD4 cell count (114 vs. 177 cells/μl, p = 0.10). At 3–12 months after starting ART, 36 patients (46%) developed virological failure (Figure 1), but these patients were distributed similarly between HIV-1 and HIV-1/2 dual infection groups (30/63 = 48% vs. 6/16 = 38%, respectively; p = 0.47). The presence or absence of virological failure did not affect the proportion of patients (14% vs. 16%, respectively; p = 0.77) that showed decreased CD4 cell counts after starting ART.

Of the 36 patients with virological failure, we performed successful resistance tests in 15 (42%). Of these, eight (60%) showed resistance to at least one antiretroviral drug: one (7%) patient was resistant to NRTIs, four (27%) to NNRTIs, and four (27%) to both. No patient showed major PI resistance.

Twelve patients were taking NNRTIs; of these, seven (58%) were resistant to NNRTIs. The most common NNRTI resistance mutation was K103N (5/12, 42%), which caused high-level resistance against efavirenz and nevirapine. One male patient with NNRTI resistance had not received NNRTI treatment but PI treatment. Among the 15 patients tested for resistance (Table 2), the most common NRTI mutation was M184V (4/15 patients, 27%). Only one patient (1/15, 7%) had thymidine analogue mutations (TAMs). All resistance mutations are shown in Table 2. The most common HIV-1 subtype was circulating recombinant form 02_AG (CRF02_AG), found in 13/15 (87%) patients. After one year of ART, no patient had switched to second-line therapy.

**Table 2:** **Distribution of resistance-associated mutations among 15 patients with viral loads > 1000 copies/ml, at 3–12 months after ART initiation**

**Patient**	**Sex**	**HIV type**	**HIV-1 subtype**	**Initial CD4**	**Baseline viral load**	**ART**	**Time from ART to re-sistance test (days)**	**NNRTI resistance**	**NRTI resistance**	**PI resistance**	**Predicted resistance to the following agents**
1	M	HIV-1	CRF02_AG	32	132,355	AZT + 3TC + EFV	319	K103N	M184M/V	None	EFV/NVP, 3TC/FTC.
2	F	HIV-1	CRF02_AG	33	61,978	AZT + 3TC + NVP	362	K103N Y188CY	None	L10I/V/F/R/Y	EFV/NVP. Resistance to most PIs when present with other mutations.
3	F	HIV-1/2	CRF02_AG	279	11,348	AZT + 3TC + IND/r	177	None	None	None	None
4	M	HIV-1/2	CRF02_AG	50	360,158	AZT + 3TC + IND/r	161	K101E	None	K20R/M/I/T/V	Intermediate NVP. Low-level ETR, RPV, EFV. Potentially low-level NFV resistance.
5	M	HIV-1	CRF02_AG	207	55,650	AZT + 3TC + EFV	114	None	None	None	None
6	M	HIV-1	CRF02_AG	46	360,978	AZT + 3TC + EFV	203	K103N K101E/K	M184MV	None	High-level 3TC/FTC.
											Low-level ddI, ABC.
											High-level EFV/NVP.
											Low-level EFV, ETR, RPV.
7	F	HIV-1	A	40	216,270	AZT + 3TC + NVP	126	K103N	M184V	L10I/V/F/R/Y	High-level 3TC/FTC.
											High-level EFV/NVP.
											Resistance to most PIs when present with other mutations.
8	F	HIV-1/2	CRF02_AG	258	46,763	AZT + 3TC + IND/r	189	None	None	None	None.
9	M	HIV-1	CRF02_AG	159	869,295	AZT + 3TC + EFV	281	None	None	None	None.
10	F	HIV-1	CRF02_AG	94	485,983	AZT + 3TC + NVP	98	V106A Y188C	None	K20R/M/I/T/V	High-level NVP, low-level EFV. Potentially low-level NFV resistance.
11	F	HIV-1	CRF02_AG	149	212,560	AZT + 3TC + NVP	344	K103N	None	None	High-level EFV/NVP.
12	M	HIV-1	CRF02_AG	107	34,698	AZT + 3TC + EFV	193	None	M184MV M184 V/I	K20R/M/I/T/V	High-level 3TC/FTC.
											Low-level ABC, ddI. Potentially low-level NFV resistance.
13	F	HIV-1	A	71	25,000,000	D4T + 3TC + NVP	98	Y181C	T69D	L10I/V/R/Y	Low-level ddI.
											High-level NVP.
											Resistance to most PIs when present with other mutations.
14	M	HIV-1	CRF02_AG	147	185	AZT + 3TC + NVP	91	None	None	L33F	Contributed resistance to FPV/r, DRV/r, LPV/r, ATV/r, TPV/r.
15	M	HIV-1	CRF02_AG	252	Un-detectable	AZT + 3TC + EFV	185	None	None	None	None.

M: Male, F: Female, AZT: Zidovudine, D4T: Stavudine, 3TC: Lamivudine, NVP: Nevirapine, EFV: Efavirenz, IND/r: Ritonavir-boosted indinavir, ABC: Abacavir, ddI: Didanosine, FPV: Fosamprenavir, DRV: Darunavir, LPV: Lopinavir, ATV: Atazanavir, TPV: Tipranavir, NFV: Nelfinavir, ETR: Etravirine, RPV: Rilpivirine.

## Discussion

The main finding of this study was that a large proportion of HIV-1 and HIV-1/2 dual infections developed virological failure (46%) after a median follow-up of six months. Furthermore, over half of these patients developed resistance - predominantly to NNRTIs. The CD4 cell count was not an effective indicator of treatment failure.

In the context of ART rollout, the most important drug resistance mutations in HIV-1 were single amino-acid mutations that conferred high-level resistance to NNRTIs. This finding was not surprising, because first-line ART and strategies for preventing mother-to-child transmission were based on NNRTIs. In this study, over half the patients with NNRTI resistance harbored the K103N mutation; thus, detection of this sentinel mutation may be useful in expanded surveillance activities for estimating the prevalence of NNRTI resistance.

Although nine patients developed clinically important mutations to either NRTIs or NNRTIs or both, none had been identified and switched to second-line therapy. The CD4 cell count was a poor predictor of treatment failure, which underscored the need for implementing virological measures.

This was the first study on HIV-1 resistance in Guinea-Bissau. Knowledge is scarce about resistance mutations in patients with HIV-1/2 dual infections. Thus, despite the small sample size, our results provided valuable information about this relatively unexplored population. Some patients with HIV-1 infections had been coincidentally treated with a first-line PI-based regimen, because they were assumed initially to be infected with HIV-1/2. This provided the opportunity to examine resistance in the context of a PI-based, rather than an NNRTI-based regimen.

Unfortunately, we could not successfully measure resistance in all patients with virological failure, due to the lack of stored plasma or difficulties with the PCR assay for some samples. The latter problem could have been due to poor sample storage in Guinea-Bissau, where electricity is intermittent. Thus, we may have underestimated the proportion of patients with resistance.

No baseline resistance tests had been performed; consequently, we could not determine whether the observed resistance was transferred or acquired. The one patient with an NNRTI-resistant virus that did not receive an NNRTI may represent a case of transmitted resistance; however, it also may represent a previous, unrecorded exposure to ART. In Senegal, the prevalence of transmitted drug resistance was 4.16% for NRTIs and 1.04% for PIs [13]. In Guinea-Conacry, the prevalence of primary resistance was 8.6% in patients naïve to ART [12]. In our study, at ART initiation, patients had low CD4 cell counts; this only occurs several years after primary infection. This finding argues against transferred resistance, because it is unlikely that this can be detected many years after a primary infection.

Studies in low- and middle-income countries have indicated, that after 12 months of ART, 82-91% of patients achieve viral suppression [17]. However, a review of 89 studies from Sub-Saharan Africa showed 78% viral suppression after six months of ART [18]. The present study, showed only 54% viral suppression; however, our shorter follow-up time made comparisons with other studies difficult.

A previous survey of acquired resistance showed that, among those experiencing therapy failure, 60-70% had developed drug resistance [17]. We identified drug resistance in 60% of patients with virological failure; this finding suggested that 40% developed treatment failure for reasons other than resistance. Thus, when viral load is used as the only indicator for switching to costlier second-line regimens, a large proportion of patients may switch unnecessarily.

The majority of patients was infected with the CRF02_AG subtype, and a minority harbored subtype A, consistent with previous studies in this region [19]. Genetic differences between subtypes might influence drug resistance pathways; therefore it may be difficult to generalize our results to studies performed in different geographic regions [20,21].

In conclusion, we found a high virological failure rate and rapid development of NNRTI resistance among patients with HIV-1 and HIV-1/2 dual infections in Guinea-Bissau. It remains to be determined whether a more robust PI-based treatment regimen might benefit this population more than NNRTIs. Further studies are warranted on drug resistance in patients with HIV-1/2 dual infections.

## Abbreviations

ART: Antiretroviral treatment
CRF02_AG: Circulating recombinant form 02_AG
IQR: Interquartile range
NNRTI: Non-nucleoside reverse transcriptase inhibitor
NRTI: Nucleoside/nucleotide reverse transcriptase inhibitors
PI: Protease inhibitor
TAMs: Thymidine analogue mutations
